# Rutin Attenuates Oxidative Stress Responses and Hepatocyte Metabolomics in β-Hydroxybutyric Acid-Induced Hepatocyte Injury in Calves

**DOI:** 10.3390/ijms26125878

**Published:** 2025-06-19

**Authors:** Kun Yang, Haixia Zhao, Min Gao, Honglian Hu, Dabiao Li

**Affiliations:** 1 College of Animal Science, Inner Mongolia Agricultural University, Hohhot 010018, China; y994454169@163.com (K.Y.); haixiazhao096@163.com (H.Z.); 2 Institute of Animal Nutrition and Feed, Inner Mongolia Academy of Agricultural & Animal Husbandry Sciences, Hohhot 010031, China; gmyh1588@126.com

**Keywords:** rutin, metabolomics, BHBA, oxidative stress

## Abstract

**:** Negative energy balance (NEB) in dairy cows induces excessive lipolysis, leading to elevated levels of β-hydroxybutyric acid (BHBA), which, when accumulated, can cause liver damage. Rutin (RT), a natural flavonoid with antioxidant and anti-inflammatory properties, has demonstrated potential hepatoprotective effects; however, its ability to mitigate BHBA-induced hepatocellular injury in calves remains unclear. This study first assessed the impact of various BHBA concentrations on oxidative stress in calf hepatocytes, then explored the protective effects and underlying mechanisms of RT, and finally employed untargeted metabolomics to further elucidate RT’s mode of action. The results showed that exposure to 1.2 mM BHBA significantly increased malondialdehyde (MDA), nitric oxide (NO) contents, and reactive oxygen species (ROS) levels, while markedly decreasing glutathione (GSH) content and catalase (CAT) activity compared with the blank control. Notably, pretreatment with 100 μg/mL RT resulted in the greatest increase in GSH contents (180%) compared to BHBA treatment alone, while 150 μg/mL RT led to the most pronounced reduction in MDA contents (220%). Furthermore, BHBA treatment significantly upregulated the expression of Kelch-like ECH-associated protein 1 (Keap1) and downregulated nuclear factor erythroid 2-related factor 2 (Nrf2), NAD(P)H quinone dehydrogenase 1 (NQO1), and heme oxygenase-1 (HO-1) at both the mRNA and protein levels. These alterations were effectively reversed by pretreatment with 100 μg/mL RT. Non-targeted metabolomics identified 1525 metabolites in total. Based on OPLS-DA, metabolites with a variable importance in projection (VIP) > 1 and *p* < 0.05 were considered significantly altered. Compared with the blank control, BHBA treatment upregulated 47 metabolites—including 8-hydroxy-2′-deoxyguanosine, 3-hydroxyisovaleric acid, and N-palmitoyl-sphingosine—and downregulated 58 metabolites, such as betaine, linolenic acid, and arachidonic acid. In contrast, RT pretreatment upregulated 207 metabolites relative to the BHBA treatment, including linolenic acid, taurocholic acid, and 4-hydroxybenzoic acid, and downregulated 126 metabolites, including 3-hydroxyisovaleric acid, 8-hydroxy-2′-deoxyguanosine, and pyruvaldehyde. Pathway enrichment analysis indicated that RT alleviated BHBA-induced hepatocyte injury primarily by modulating the fatty acid degradation pathway. In summary, RT mitigated BHBA-induced oxidative stress in calf hepatocytes by regulating the Keap1/Nrf2 signaling pathway and further exerted protective effects through metabolic reprogramming.

## 1. Introduction

Periparturient cows are highly susceptible to negative energy balance (NEB), which triggers excessive lipolysis and leads to the accumulation of short-chain fatty acids—such as non-esterified fatty acids and β-hydroxybutyric acid (BHBA)—in the liver. The impaired metabolism of these fatty acids results in liver injury [1]. Studies have demonstrated a positive correlation between blood BHBA levels and oxidative stress markers, including glutathione, catalase, and superoxide dismutase, in ketotic cows. Notably, the incidence of ketosis in dairy cows currently reaches approximately 15% or higher, posing a significant threat to dairy production efficiency [2]. Moreover, elevated BHBA concentrations disrupt blood lipid metabolism indicators [3]. Zhou et al. [4] reported that high BHBA levels increase hepatic triglyceride (TG) content and inhibit cholesterol synthesis, thereby causing lipid metabolism disorders in dairy cows. Similarly, Deng et al. [5] found that BHBA activates the AMP-activated protein kinase (AMPK) signaling pathway, regulating genes involved in lipid synthesis and oxidation. Collectively, these findings indicate that excessive BHBA induces oxidative stress and lipid metabolism disturbances in dairy cows.

Rutin (RT) is a natural flavonoid with diverse biological activities, including anti-inflammatory, antioxidant, antibacterial, and antitumor effects. Dietary supplementation with RT has been shown to alleviate oxidative stress by reducing hydrogen peroxide (H_2_O_2_) and malondialdehyde (MDA) contents in sheep mammary tissue, while upregulating mRNA expression of nuclear factor erythroid 2-related factor 2 (Nrf2) and heme oxygenase-1 (HO-1) [6]. Naderi et al. [7] reported that RT mitigated perfluorooctanoic acid (PFOA)-induced liver injury in rats by decreasing MDA and glutathione (GSH) contents, reducing superoxide dismutase (SOD) activity, and alleviating oxidative stress and mitochondrial dysfunction. Peng et al. [8] demonstrated that RT significantly lowered serum TG content and alleviated hepatic lipid accumulation in rats, improving lipid metabolism disorders. Additionally, Ma et al. [9] found that RT reduced MDA and total cholesterol (TC) contents in mouse testicular tissue, mitigating oxidative stress and lipid metabolism impairment. Similarly, recent studies have shown that the flavonoid prunin enhances antioxidant capacity by increasing activities of enzymes such as SOD and catalase (CAT) [10]. However, the potential of RT to counteract BHBA-induced hepatic oxidative stress in dairy cows remains largely unexplored. We hypothesized that RT could attenuate oxidative stress in hepatocytes exposed to high BHBA concentrations. This study aimed to evaluate the protective effects of RT against BHBA-induced oxidative stress in primary calf hepatocytes and to elucidate the underlying mechanisms via the Keap1/Nrf2 signaling pathway and untargeted metabolomics.

## 2. Results

### 2.1. Effect of BHBA on Oxidative Stress Injury in Calf Hepatocytes

**Figure 1:**
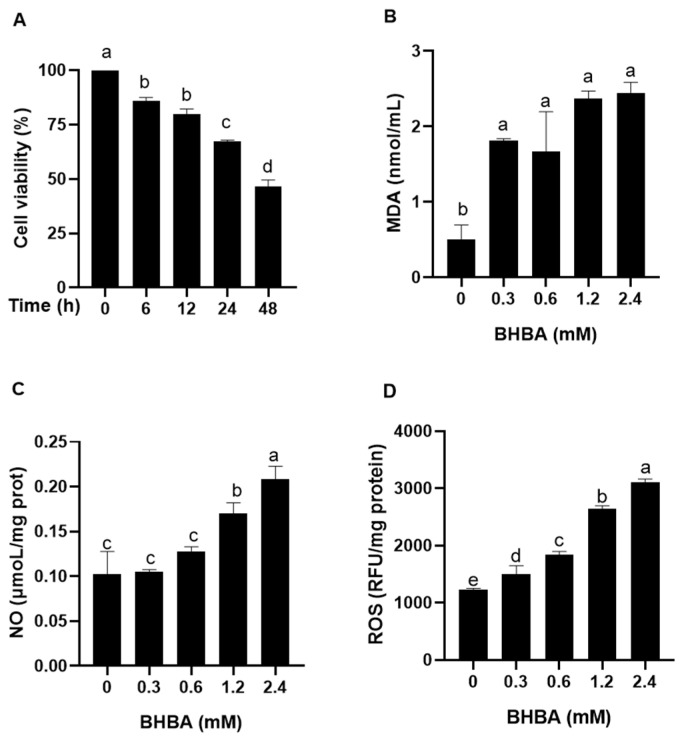
Effect of BHBA on hepatocyte viability, and oxidative stress injury in calves. (**A**) The cell viability; cells were challenged with 1.2 mM BHBA for different times (0, 6, 12, 24, 48 h), respectively. Cell viability was measured using the CCK-8 assay. (**B**–**D**) Effect of BHBA on oxidative stress indices. Contents of MDA, NO, and ROS levels in hepatocytes were measured using assay kits. Cells were treated with different concentrations of BHBA (0, 0.3, 0.6, 1.2, and 2.4 mM) for 24 h. The ROS levels were measured in terms of relative fluorescence units (RFUs) at excitation and emission wavelengths of 485 nm and 535 nm. Each experiment was repeated at least six times. The data are shown as the mean ± SEM. The values with different lowercase letters are significantly different (*p* < 0.05).

As shown in Figure 1A, there was no significant difference in calf hepatocyte viability between 6 h and 12 h of BHBA treatment (*p* > 0.05). However, at 24 h, hepatocyte viability significantly decreased compared to earlier time points, though it remained higher than at 48 h (*p* < 0.05). Both 1.2 and 2.4 mM BHBA significantly increased MDA and NO contents, as well as the ROS levels in calf hepatocytes (Figure 1B–D, *p* < 0.05). Notably, no significant difference was observed in MDA content (Figure 1D, *p* > 0.05). Based on these findings, 1.2 mM BHBA was selected as the model concentration for subsequent experiments.

### 2.2. Effect of RT on BHBA-Induced Oxidative Stress in Hepatocytes

**Figure 2:**
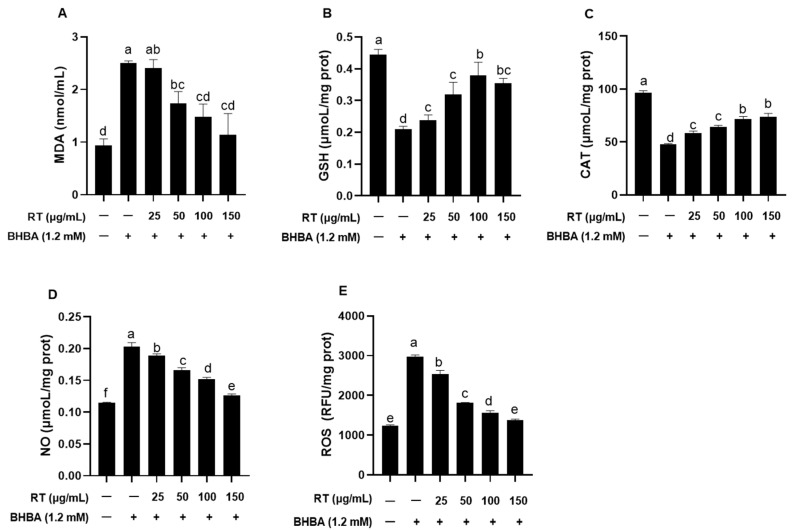
Effect of RT on BHBA-induced oxidative stress in calf hepatocytes. (**A**–**E**) MDA, GSH, and NO contents, ROS levels, and CAT activity were measured using assay kits. Cells were pretreated with different concentrations of RT (0, 25, 50, 100, 150 μg/mL) for 24 h and then challenged with 1.2 mM BHBA for 24 h. The ROS levels were measured in terms of relative fluorescence units (RFUs) at excitation and emission wavelengths of 485 nm and 535 nm. Each experiment was repeated at least six times. The data are shown as the mean ± SEM. The values with different lowercase letters are significantly different (*p* < 0.05).

As shown in Figure 2, BHBA significantly increased MDA and NO contents as well as ROS levels, while significantly reducing GSH content and CAT activity in hepatocytes compared to the blank control. Pretreatment with different concentrations of RT effectively reversed these changes, with 100 and 150 μg/mL showing the most pronounced protective effects (Figure 2A–E, *p* < 0.05). Notably, pretreatment with 100 μg/mL RT resulted in the greatest increase in GSH content (180%) compared to BHBA treatment alone, whereas 150 μg/mL RT led to the most pronounced reduction in MDA content (220%). No significant differences were observed in MDA and GSH contents or CAT activity between the 100 μg/mL and 150 μg/mL RT treatment (Figure 2A–C, *p* > 0.05).

### 2.3. Effect of RT on Keap1-Nrf2 Signaling Pathway in Hepatocytes

**Figure 3:**
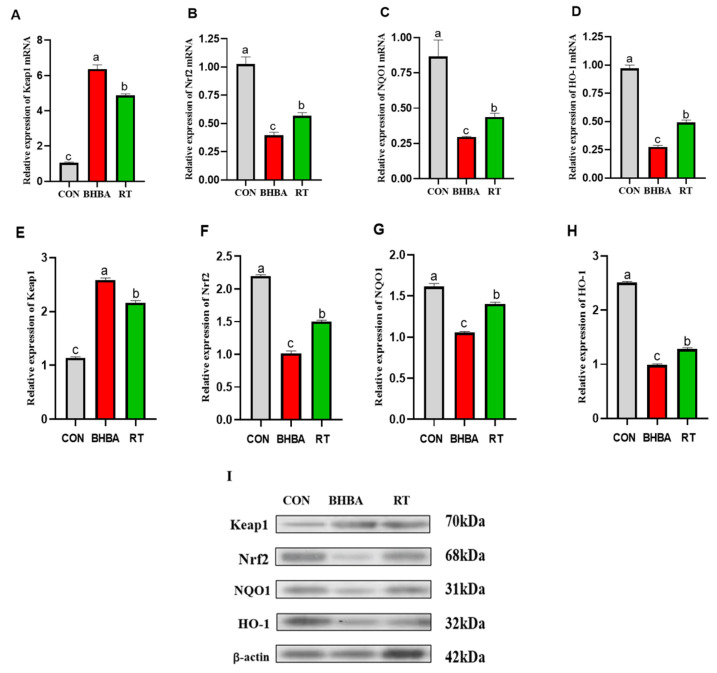
RT alleviated the oxidative stress induced by BHBA in calf hepatocytes by activating Keap1-Nrf2 signaling pathway. (**A**–**D**) Relative mRNA expression levels of Keap1, Nrf2, NQO1, and HO-1 were detected using RT-qPCR with β-actin used as an endogenous control. (**E**–**I**) Expression levels of proteins Keap1, Nrf2, NQO1, and HO-1 were detected using Western blotting, and β-actin was used as a control. Blank control (CON, well-grown hepatocytes, untreated), BHBA treatment (cells were treated with 1.2 mM BHBA for 24 h), RT treatment (cells were pretreated with 100 μg/mL RT for 24 h and then challenged with 1.2 mM BHBA for 24 h). Each experiment was repeated at least six times. The data are shown as the mean ± SEM. The values with different lowercase letters are significantly different (*p* < 0.05).

As shown in Figure 3, BHBA significantly upregulated the mRNA and protein expression of Keap1, while markedly downregulating the expression of Nrf2, NQO1, and HO-1 in hepatocytes (*p* < 0.05). In contrast, RT treatment effectively reversed these alterations (*p* < 0.05).

### 2.4. Metabolome Analysis

#### 2.4.1. Multivariate Statistical Analysis

**Figure 4:**
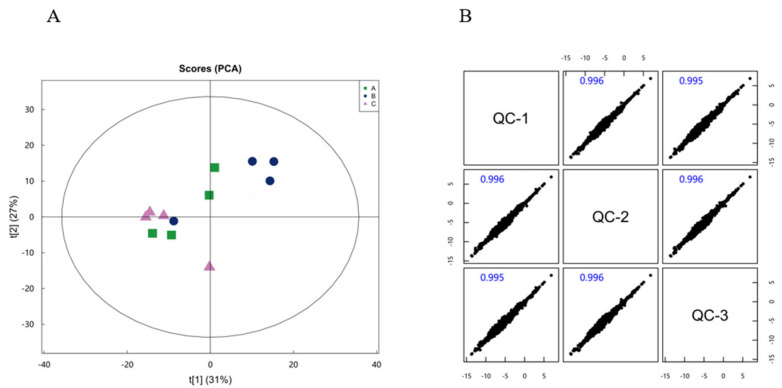

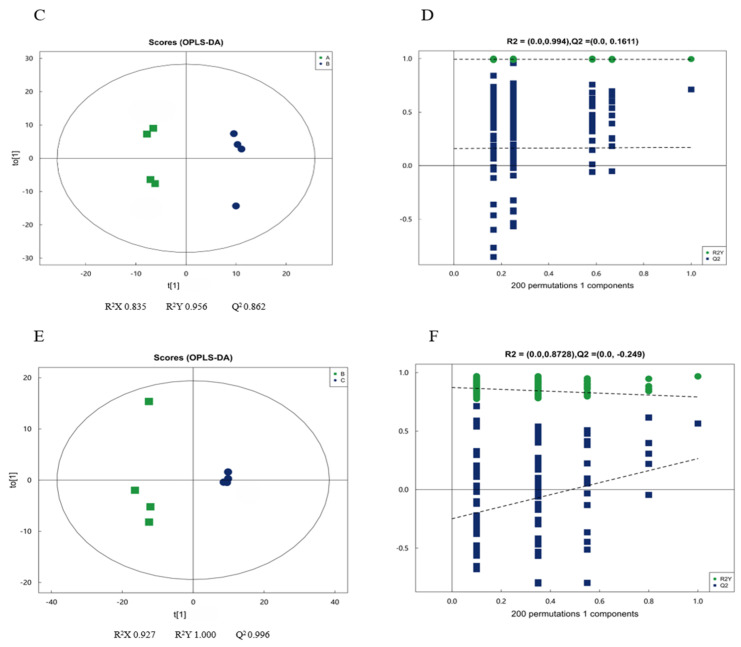
Differential metabolites analysis based on calf hepatocytes’ metabolomics. (**A**) Plot of ALL mode PCA score. (**B**) Scatter Plot Matrix. (**C**) Control blank vs. BHBA treatment orthogonal projections to latent structures discriminant analysis (OPLS-DA) test in the ALL mode. (**D**) Control blank vs. BHBA treatment orthogonal partial least squares (PLS-DA) test in the ALL mode. (**E**) BHBA treatment vs. RT treatment orthogonal projections to latent structures discriminant analysis (OPLS-DA) test in the ALL mode. (**F**) BHBA treatment vs. RT treatment orthogonal partial least squares (PLS-DA) test in the ALL mode. Blank control (well-grown hepatocytes, untreated), BHBA treatment (cells were treated with 1.2mM BHBA for 24 h), RT treatment (cells were pretreated with 100 μg/mL RT for 24 h and then challenged with 1.2 mM BHBA for 24 h). Each experiment was repeated at least four times.

Principal component analysis (PCA; Figure 4A,B) revealed clear separation among the three groups, with the first two components, PC1 (31%) and PC2 (27%), explaining 58% of the total variance. All sample confidence intervals fell within the 95% limit, confirming data reliability after dimensionality reduction. Orthogonal partial least squares discriminant analysis (OPLS-DA; Figure 4C–F) further validated group discrimination, with R^2^Y and Q^2^ values exceeding 0.5, indicating a robust and predictive model. Permutation testing of the OPLS-DA model yielded R^2^ values of 0.994 and 0.873 and Q^2^ values of 0.161 and −0.249, confirming that the model’s predictive performance was reliable for subsequent metabolomic analyses.

#### 2.4.2. Screening and Identification of Differential Metabolites

**Figure 5:**
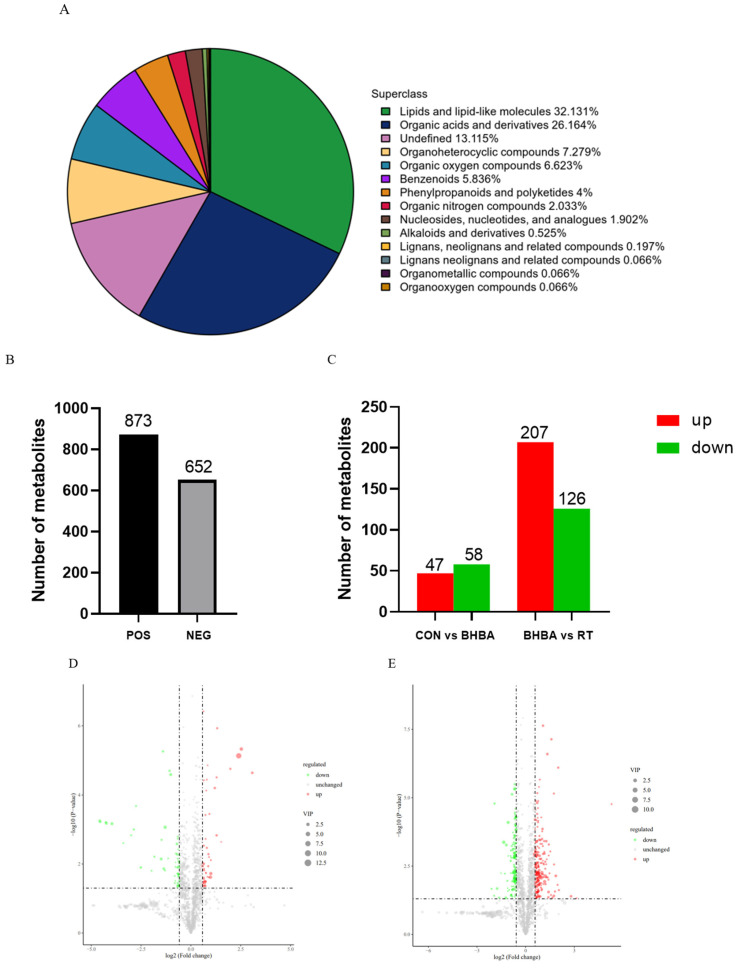
Differential metabolites analysis based on calf hepatocyte metabolomics. (**A**) Metabolite classification map. (**B**) The number of metabolites in the positive and negative ion modes. (**C**) The number of differential metabolites among blank control (CON), BHBA and RT treatment. Volcano plot of differential metabolites in total ion mode, (**D**) CON vs. BHBA, (**E**) BHBA vs. RT. Blank control (well-grown hepatocytes, untreated), BHBA treatment (cells were treated with 1.2 mM BHBA for 24 h), RT treatment (cells were pretreated with 100 μg/mL RT for 24 h and then challenged with 1.2 mM BHBA for 24 h).

**Figure 6:**
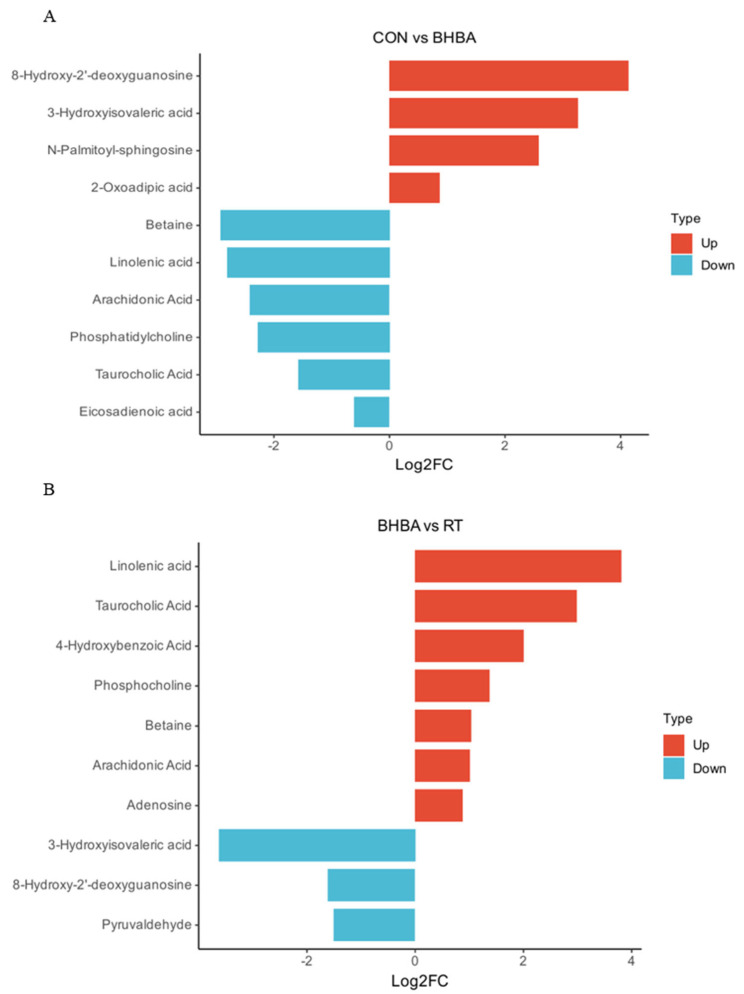
Enrichment analysis of the top 10 pathways for differential metabolites. (**A**) CON vs. BHBA, (**B**) BHBA vs. RT. Blank control (CON, well-grown hepatocytes, untreated), BHBA treatment (cells were treated with 1.2 mM BHBA for 24 h), RT treatment (cells were pretreated with 100 μg/mL RT for 24 h and then challenged with 1.2 mM BHBA for 24 h).

Non-targeted metabolomics identified a total of 1525 metabolites, primarily comprising lipids and lipid-like molecules (32.13%), organic acids and derivatives (26.16%), and organic heterocyclic compounds (6.62%). Among these, 873 metabolites were detected in positive ion mode and 652 in negative ion mode (Figure 5A,B). Metabolites were further screened using the OPLS-DA model, with VIP > 1 and *p* < 0.05 (*t*-test) as the selection criteria. A comparison between the blank control and BHBA treatment identified 47 upregulated and 58 downregulated metabolites, while comparison between the BHBA and RT treatments revealed 207 upregulated and 126 downregulated metabolites (Figure 5C). These differential metabolites were further visualized using volcano plots (Figure 5D,E). The top 10 most prominent metabolites were selected for further analysis. As shown in Figure 6A, compared to the blank control, 8-hydroxy-2′-deoxyguanosine, 3-hydroxyisovaleric acid, N-palmitoyl-sphingosine, and 2-oxoadipic acid were significantly upregulated, whereas betaine, linolenic acid, arachidonic acid, phosphatidylcholine, taurocholic acid, and eicosadienoic acid were downregulated. In Figure 6B, RT treatment upregulated linolenic acid, taurocholic acid, 4-hydroxybenzoic acid, phosphocholine, betaine, arachidonic acid, and adenosine, while downregulating 3-hydroxyisovaleric acid, 8-hydroxy-2′-deoxyguanosine, and pyruvaldehyde compared to the BHBA treatment.

#### 2.4.3. Differential Metabolite Pathway Enrichment Analysis

**Figure 7:**
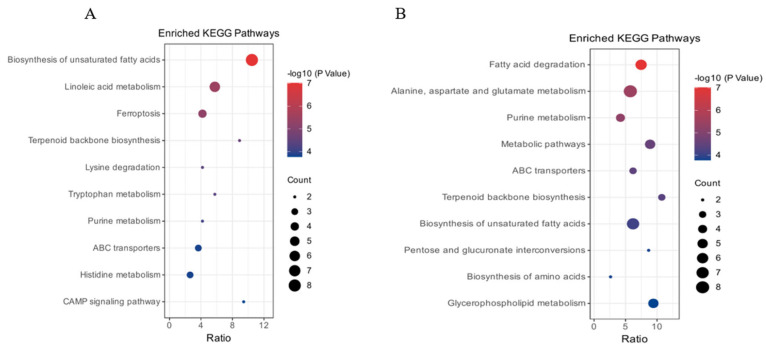
Screening of the top 10 dominant differential metabolites in total ion mode. (**A**) CON vs. BHBA, (**B**) BHBA vs. RT. Blank control (CON, well-grown hepatocytes, untreated), BHBA treatment (cells were treated with 1.2 mM BHBA for 24 h), RT treatment (cells were pretreated with 100 μg/mL RT for 24 h and then challenged with 1.2 mM BHBA for 24 h).

Pathway enrichment analysis of differential metabolites based on the total ion model is shown in Figure 7A. Compared to the blank control, BHBA treatment primarily enriched pathways related to biosynthesis of unsaturated fatty acids, linoleic acid metabolism, ferroptosis, ABC transporters, and histidine metabolism. As shown in Figure 7B, compared to the BHBA treatment, RT treatment mainly enriched pathways involved in fatty acid degradation, alanine, aspartate and glutamate metabolism, biosynthesis of unsaturated fatty acids, and general metabolic pathways.

## 3. Discussion

Periparturient dairy cows frequently experience NEB due to insufficient energy intake relative to production demands [11]. Prolonged NEB leads to excessive mobilization of adipose tissue and elevated production of ketone bodies, particularly BHBA. Excessive accumulation of BHBA in the liver via circulation can cause hepatic injury and contribute to the development of ketosis in dairy cows [12,13]. Additionally, high BHBA concentrations have been shown to induce oxidative stress in bovine tissues [3]. RT, a natural flavonoid, possesses potent anti-inflammatory and antioxidant activities. Our previous study demonstrated that RT alleviates BHBA-induced lipid metabolism disorders and inflammatory injury in calf hepatocytes [14]. The present study further confirms that RT mitigates BHBA-induced oxidative stress through modulation of the Keap1/Nrf2 signaling pathway, with underlying mechanisms elucidated via untargeted metabolomics analysis.

In this study, hepatocyte viability dropped below 50% following 48 h of exposure to 1.2 mM BHBA, rendering it unsuitable for subsequent experiments; therefore, a 24 h exposure duration was selected. Li et al. [15] reported that 2.4 mM BHBA significantly increased MDA content and ROS levels in bovine mammary epithelial cells after 24 h, inducing oxidative stress. Similarly, Mirzaei et al. [16] demonstrated in vivo that blood BHBA levels in periparturient cows were positively correlated with MDA content, a key oxidative stress marker. Mohsin et al. [17] further showed that increasing BHBA concentration and exposure time in calf hepatocytes led to a progressive rise in MDA content and a concomitant decline in SOD and CAT activities. Consistent with these findings, our results showed that both 1.2 mM and 2.4 mM BHBA significantly elevated MDA and NO contents as well as ROS levels in calf hepatocytes after 24 h, confirming that high concentrations of BHBA induce oxidative stress in these cells. To assess whether RT mitigates BHBA-induced oxidative stress in calf hepatocytes, cells were pretreated with various concentrations of RT. Compared with BHBA treatment alone, RT significantly reduced MDA, NO, and ROS levels, while enhancing CAT activity and GSH content. Among the tested doses, 100 and 150 μg/mL RT exhibited the most pronounced protective effects. These findings are supported by Saafan et al. [18], who reported that oral administration of 50 or 100 mg/kg RT attenuated D-galactose-induced oxidative stress in rat liver by enhancing antioxidant enzymes such as SOD and glutathione peroxidase (GSH-Px). Similarly, Iqbal et al. [19] showed that 100 mg/kg RT significantly decreased MDA content and increased CAT activity, thereby alleviating oxidative stress in rats. Orzuna et al. [20] further reported in a meta-analysis that flavonoids reduce serum MDA content, mitigating oxidative stress in dairy and beef cattle. In line with these results, Guo et al. [21] demonstrated that oxidative stress markers—T-AOC, SOD, GSH-Px, and MDA—were significantly altered in periparturient cows experiencing oxidative stress. Collectively, our findings confirm that RT effectively alleviates BHBA-induced oxidative stress in calf hepatocytes by decreasing oxidative damage markers and enhancing antioxidant defense mechanisms. Previous studies have shown that the antioxidant vitamin E alleviates hydrogen peroxide-induced oxidative stress in sheep hepatocytes by reducing intracellular ROS level [22], while silymarin mitigates oxidative damage in rat liver tissue by decreasing MDA content [23]. Therefore, future studies should include a positive control treatment (e.g., vitamin E) to enable a comparative evaluation of the protective effects and underlying mechanisms of RT against oxidative stress in calf hepatocytes. A key limitation of the current study is the lack of in vivo validation; only an in vitro hepatocyte model was employed to assess the protective effects of RT, which may not fully represent the complexity of hepatic injury in ketotic cows.

The Keap1/Nrf2 signaling pathway is widely recognized as a classical antioxidant defense mechanism. Nrf2, a key transcription factor, orchestrates the cellular response to oxidative stress. Under basal conditions, Nrf2 is retained in the cytoplasm by its repressor Keap1 [24]. Upon oxidative challenge, Nrf2 dissociates from Keap1, translocates into the nucleus, and binds to antioxidant response elements (AREs), thereby inducing the expression of antioxidant enzymes such as HO-1, NQO1, and CAT [24]. Hussein et al. [25] demonstrated that the flavonoid quercetin alleviated oxidative liver injury in rats by downregulating Keap1 and upregulating Nrf2 and HO-1 mRNA levels. Similarly, bamboo leaf flavonoids were shown to attenuate oxidative stress in broiler liver by modulating the expression of Keap1, NQO1, and HO-1 [26]. Singh et al. [27] reported that RT ameliorated tert-butyl hydroperoxide-induced oxidative damage in mouse liver via activation of the Keap1/Nrf2 pathway. In the present study, to determine whether RT exerts similar protective effects in calf hepatocytes, cells were pretreated with 100 μg/mL RT prior to exposure to 1.2 mM BHBA. RT treatment significantly upregulated the mRNA and protein levels of Nrf2, NQO1, and HO-1, while markedly downregulating Keap1 expression compared to BHBA treatment alone, suggesting that RT confers protection through activation of the Keap1/Nrf2 signaling pathway.

To further elucidate the mechanisms by which RT attenuates BHBA-induced hepatocyte injury, untargeted metabolomics analysis was performed on calf hepatocytes. A total of 1525 metabolites were identified in total ion mode. Differential metabolites were screened using OPLS-DA, with VIP > 1 and *p* < 0.05. The top 10 significantly altered metabolites were further analyzed. Among them, 8-hydroxy-2′-deoxyguanosine is a well-established biomarker of oxidative stress [28,29]; 3-hydroxyisovaleric acid, a leucine catabolite [30], has been associated with interleukin-17 (IL-17) production [31]; ceramide, a bioactive sphingolipid, contributes to inflammation and metabolic dysfunction through the formation of N-palmitoyl-sphingosine [32]; and 2-oxoadipic acid, a lysine metabolism intermediate, has been implicated in oxidative damage when excessively accumulated [33,34]. In this study, exposure to high concentrations of BHBA significantly increased the levels of these metabolites in calf hepatocytes. These results suggest that BHBA induces oxidative stress and impairs both amino acid and lipid metabolism, contributing to hepatocellular injury in calves. Betaine has been shown to alleviate oxidative stress in the liver tissues of dairy cows [35]. Linolenic acid, a polyunsaturated fatty acid, enhances hepatocellular antioxidant capacity following catabolism [36]. Reduced levels of arachidonic acid and taurocholic acid are indicative of impaired energy and lipid metabolism in hepatic tissues [37]. Moreover, phosphatidylcholine plays a protective role by preventing excessive lipid accumulation in hepatocytes and reducing lipotoxicity [38]. In the present study, high BHBA exposure significantly decreased the levels of these metabolites, further supporting the hypothesis that BHBA disrupts lipid homeostasis and promotes oxidative stress in calf hepatocytes.

Conversely, pretreatment with 100 μg/mL RT upregulated beneficial metabolites—including linolenic acid, taurocholic acid, 4-hydroxybenzoic acid, phosphocholine, betaine, arachidonic acid, and adenosine—while downregulating 3-hydroxyisovaleric acid, 8-hydroxy-2′-deoxyguanosine, and pyruvaldehyde. Li et al. [39] reported that the flavonoid astragalin ameliorated hepatic lipid metabolism disorders in a mouse model of nonalcoholic fatty liver disease by increasing linolenic acid levels in liver tissue. Similarly, Song et al. [40] demonstrated that taurocholic acid alleviated cholestatic liver injury in mice through activation of the Nrf2 signaling pathway. Winter et al. [41] found that 4-hydroxybenzoic acid attenuated H_2_O_2_-induced oxidative stress in neuronal cells by reducing nitric oxide levels. Moreover, Abenko et al. [42] showed that chamomile flavonoids increased hepatic phosphocholine content and mitigated carbon tetrachloride-induced lipid metabolic injury in rat liver. Váli et al. [43] reported that betaine alleviated oxidative stress in rat liver by increasing hepatic GSH-Px and SOD activities. Ji et al. [44] demonstrated that the flavonoid kaempferol enhanced systemic antioxidant capacity by upregulating SOD and GSH-Px activities in mouse muscle tissue, along with elevating glycogen and adenylate levels in liver and skeletal muscle. In the present study, RT increased the levels of these metabolites in calf hepatocytes, consistent with the above findings. Leipnitz et al. [45] showed that 3-hydroxyisovaleric acid induced oxidative protein damage in rat brain by promoting carbonyl formation and sulfhydryl oxidation. Elevated 8-hydroxy-2′-deoxyguanosine levels are widely recognized as biomarkers of oxidative stress. Hesperidin, a flavonoid, was shown to significantly reduce the expression of 8-hydroxy-2′-deoxyguanosine, NF-κB, and MDA in rat colonic tissues, thereby alleviating colitis-induced oxidative damage [46]. Pyruvaldehyde, a glycolytic and lipid metabolism by-product derived from glycerol and ketone bodies, has been linked to hyperglycemia, hyperlipidemia, and obesity, and contributes to lipid metabolism disorders and oxidative stress in liver tissues [47]. In the present study, RT significantly reduced the levels of these harmful metabolites in BHBA-induced calf hepatocytes, further confirming its protective role against BHBA-induced hepatic oxidative and lipid metabolic injury. Zhang et al. [48] demonstrated that the flavonoid quercetin alleviated acrylamide-induced oxidative stress and lipid metabolism disorders in rat liver by modulating fatty acid metabolites such as taurine and phosphatidylcholine, as revealed by metabolomics analysis. Similarly, Wang et al. [49] reported that the flavonoid chrysin mitigated liver injury in NAFLD rats by regulating cholesterol degradation and metabolism pathways. In the present study, pathway enrichment analysis of differential metabolites showed that those altered in the BHBA treatment were primarily involved in the biosynthesis of unsaturated fatty acids, whereas metabolites in the RT treatment were mainly enriched in the fatty acid degradation pathway. A limitation of this study is the relatively small number of biological replicates in the non-targeted metabolomics analysis; thus, further validation of key differential metabolites is needed in future research.

## 4. Materials and Methods

### 4.1. Isolation and Culture of Primary Calf Hepatocytes

Newborn healthy Holstein female calves were fasted, and primary hepatocytes were isolated using a two-step perfusion method within 24 h. Briefly, calves were anesthetized with thiamylal sodium (50 mg/kg) and administered an intravenous injection of heparin (1500 IU/kg). The caudate lobe of the liver was rapidly excised, and blood residues were removed with sterile saline. The lobe was then perfused with Solution A (140 mM NaCl, 10 mM HEPES, 6.7 mM KCl, 0.5 mM EDTA, and 2.5 mM glucose, pH 7.4), pre-warmed to 37 °C, at a flow rate of 50 mL/min for approximately 15 min. Subsequently, perfusion continued with Solution B (140 mM NaCl, 30 mM HEPES, 6.7 mM KCl, 5 mM CaCl_2_, and 2.5 mM glucose, pH 7.4) at the same flow rate until the effluent became clear, lasting approximately 10 min. The liver was then perfused with Solution B containing 0.0002 g/mL collagenase IV (Type IV Collagenase, Gibco, Waltham, MA, USA) through the blood vessels until the solution became turbid. After digestion, the caudate lobe was carefully dissected through the peritoneum to remove blood vessels, fat, and undigested tissues, then immersed in fetal bovine serum (FBS; Hyclone Laboratories, Logan, UT, USA) to terminate digestion. The cell suspension was subsequently filtered twice through 100-mesh and 200-mesh filters. Hepatocytes and their termination solution were transferred into 50 mL centrifuge tubes and centrifuged at 800× *g* for 10 min. After aspirating the supernatant, the cell pellet was washed three times with RPMI-1640 medium (Hyclone Laboratories). The cell suspension was then transferred to a large beaker, and the density was adjusted to 2 × 10^6^ cells/mL using RPMI-1640 adherence medium (Sigma-Aldrich Co., St. Louis, MO, USA), which was prepared by supplementing 250 mL of RPMI-1640 medium with 10% FBS, 1 mM bovine insulin (230 μL), 1 mM dexamethasone (8 μL), 5 mg/mL vitamin C (4 μL), and penicillin-streptomycin at a final concentration of 1%. The cell suspension was gently pipetted to ensure uniform resuspension before being inoculated into 6-well plates (2 mL per well) and 96-well plates. The plates were incubated under adherent culture conditions at 37 °C with 5% CO_2_ for 4 h. After adherence, the culture medium was replaced with RPMI-1640 complete medium (RPMI-1640 medium supplemented with 10% FBS), and the medium was subsequently changed every 24 h.

### 4.2. Cell Grouping and Treatment

Firstly, healthy hepatocytes were cultured and exposed to varying concentrations of BHBA (0, 0.3, 0.6, 1.2, and 2.4 mM) for 24 h to determine the optimal concentration for inducing hepatocyte injury. Subsequently, well-grown hepatocytes were pretreated with different concentrations of RT (0, 25, 50, 100, and 150 μg/mL) for 24 h, followed by treatment with 1.2 mM BHBA for an additional 24 h. The effects of RT on BHBA-induced oxidative stress in hepatocytes were then evaluated. Finally, the hepatocytes were divided into three groups: (1) Blank control: untreated, healthy hepatocytes; (2) BHBA treatment: hepatocytes exposed to 1.2 mM BHBA for 24 h; (3) RT treatment: hepatocytes pretreated with 100 μg/mL RT for 24 h, followed by treatment with 1.2 mM BHBA for another 24 h. This experimental setup aimed to investigate the protective effects of RT on BHBA-induced hepatocyte injury, specifically through its regulation of the Keap1/Nrf2 signaling pathway, while also exploring the underlying mechanisms using metabolomic analysis. Each treatment group had six replicates.

### 4.3. Cell Viability Assay

Well-grown hepatocytes in 96-well plates were selected and replaced with RPMI-1640 complete medium containing a final concentration of 1.2 mM BHBA. The cells were then cultured for 0, 6, 12, 24, and 48 h. After incubation, 10 μL of CCK-8 reagent (Beyotime, Shanghai, China) was added to each well and incubated for an additional 3 h. Absorbance at 450 nm was measured using a microplate reader (Thermo Fisher Scientific, Waltham, MA, USA).

### 4.4. Measurement of Oxidative Stress Indicators

The hepatocytes were harvested after incubation with different concentrations of BHBA (with or without RT) for 24 h and washed thrice with phosphate-buffered saline (PBS). The cell sediment was collected, and reactive oxygen species (ROS) levels were measured strictly according to the kit instructions (Angle Gene Co., Ltd., Nanjing, China). The cells were then lysed using P0013D (Beyotime, Nanjing, China), and the lysate was centrifuged at 12,000× *g* for 5 min at 4 °C. The resulting supernatant was used to determine MDA, NO, and GSH contents, as well as CAT activity, using the corresponding biochemical kits (Angle Gene Co., Ltd., Nanjing, China) following the manufacturer’s protocols.

### 4.5. RNA Extraction and Real-Time PCR

At the end of the experiment, the cell precipitates were collected, and the cells were lysed by adding 1 mL of TRIzol reagent (TaKaRa Biotechnology Co., Ltd., Tokyo, Japan) respectively. RNA was extracted following the steps of the RNA extraction kit and RNA concentration was determined using a K5500 Micro-Spectrophotometer (Beijing Kaiao Technology Development Co., Ltd., Beijing, China). The cDNA was synthesized according to the steps of the reverse transcription kit (TaKaRa Biotechnology, RR047A, Dalian, China), and primer design for real-time fluorescence quantitative PCR was performed using PrimerPremier 6.0 and Beacon designer 7.8 software. The primer sequences are shown in Table 1. RT-qPCR was performed using MonclerTM ChemoHS qPCR Mix (SYBR green; Roche, Basel, Switzerland) and a CFX 96 real-time PCR system (Bio-Rad, Hercules, CA, USA). Expression of all target genes was determined using the endogenous gene β-actin as a control. The 2^−∆∆CT^ method was used to calculate the relative expression of the target genes.

### 4.6. Western Blotting

After collecting the cell sediment, total protein from primary calf hepatocytes was extracted following the protocol of the total protein extraction kit (Sangon Biotech Co., Ltd., Shanghai, China). The extracted protein was then quantified using the BCA quantification kit (Sangon Biotech Co., Ltd., Shanghai, China). A 5% stacking gel was prepared, and 60 μg of total protein was loaded into each well for separation by SDS-PAGE electrophoresis. After protein separation, the membrane was transferred and washed twice with Tris-HCl buffer solution (T-TBS). It was then blocked in TBS containing 5% skimmed milk powder for 1 h at room temperature. Subsequently, the PVDF membrane was incubated overnight at 4 °C with diluted primary antibodies (Keap1, Nrf2, NQO1, HO-1, and β-actin), followed by washing with TBS. The PVDF membrane hybridized with the primary antibody was incubated with a diluted secondary antibody at room temperature for 1 h, followed by rinsing with TBS solution. Immunodetection was performed using an enhanced chemiluminescence (ECL) solution (Pierce Biotechnology Inc., Chicago, IL, USA). The optical density of the bands was analyzed using National Institutes of Health (Quantity One 1-D) Quantity One software (4.6.8).

### 4.7. Metabolomics Data Analysis

Non-targeted metabolomics was performed on 12 cell samples, including 4 from the blank control, 4 from the BHBA treatment, and 4 from the RT treatment. An appropriate amount of each sample was added to a pre-cooled methanol/acetonitrile/water solution (2:2:1, *v*/*v*), followed by vortex mixing, low-temperature sonication for 30 min, incubation at −20 °C for 10 min, and centrifugation at 14,000× *g* for 20 min at 4 °C. The supernatant was dried under vacuum, and 100 μL of aqueous acetonitrile (acetonitrile:water = 1:1, *v*/*v*) was added for mass spectrometry analysis. The mixture was vortexed and centrifuged at 14,000× *g* for 15 min at 4 °C, and the resulting supernatant was collected for analysis. Sample separation was performed using an Agilent 1290 Infinity LC ultra-high-performance liquid chromatography (UHPLC, 1290 Infinity II) system. The samples were separated using UHPLC and analyzed on a Triple TOF 6600 mass spectrometer (AB SCIEX, Triple TOF 6600+). Peak alignment, retention time correction, and peak area extraction were performed using XCMS software (XCMS in MetaboAnalyst). The extracted data underwent metabolite structure identification, preprocessing, quality assessment, and subsequent data analysis. Orthogonal partial least squares discriminant analysis (OPLS-DA) was conducted using Ropls software (1.40.0) to identify differential metabolites based on the criteria of *p*-value < 0.05, variable importance in projection (VIP > 1), and fold change (FC > 2). Functional pathway enrichment of the identified differential metabolites was performed using MetaboAnalyst (5.0), with significant enrichment defined as *p*-value < 0.05.

### 4.8. Statistical Analysis

All data were analyzed using SPSS 22.0 (SPSS Inc., Chicago, IL, USA). The normality of all parameters was assessed using the Shapiro–Wilk test. Data were expressed as mean ± standard error of the mean (SEM). For normally distributed data, statistical comparisons were performed using a two-tailed unpaired Student’s *t*-test or one-way ANOVA followed by Bonferroni correction. For non-normally distributed data, the Mann–Whitney U test was used. Statistical significance was set at *p* < 0.05.

## 5. Conclusions

In summary, RT alleviated BHBA-induced oxidative stress in calf hepatocytes by modulating the Keap1/Nrf2 signaling pathway. Non-targeted metabolomics further revealed that RT attenuated oxidative stress and lipid metabolism disorders by promoting fatty acid degradation, as evidenced by the upregulation of metabolites such as linolenic acid and taurocholic acid, and the downregulation of 3-hydroxyisovaleric acid and 8-hydroxy-2′-deoxyguanosine. These findings provide a theoretical basis for improving dairy cow health during early lactation and highlight the potential of RT as a functional feed additive in the dairy industry.

## Figures and Tables

**Table 1:** Primer sequences (F = forward; R = reverse).

Gene	Accession No.	Primer Sequence (5′ to 3′)	Length (bp)
ACTB	NM_280979	F: GCAAATGCTTCTAGGCGGAC	203
		R: ATGCTCGATCCAACCGACTG	
Keap1	NM_3476823	F: AGAGAAACGAGTGGCGGATG	159
		R: ACGTCCACGTTTCTGTCTCC	
Nrf2	NM_4092041	F: AGCCTCAAAGCACCGTCCTC	171
		R: TGTCAATCAAATCCATGTCCTGC	
NQO1	NM_4938920	F: GATCGTACTGGCCCACTCAG	106
		R: CCGGGGTCCTTCAGTTTACC	
HO-1	NM_3950281	F: TGAGCTGACCCAAGAAGGTT	135
		R: AGTGTAGACGGGGTTCTCCTT	

## Data Availability

The data presented in this study are available within the article.
